# Huoxue Wentong Formula ameliorates myocardial infarction in rats through inhibiting CaMKII oxidation and phosphorylation

**DOI:** 10.1186/s13020-020-0285-2

**Published:** 2020-01-10

**Authors:** Tiantian Liu, Qingqing Wang, Kuiwu Yao

**Affiliations:** Department of Cardiology, Guang’anmen Hospital, China Academy of Chinese Medical Sciences, No. 5 Beixian’ge, District of Xi Cheng, Beijing, 100053 China

**Keywords:** HXWTF, CaMKII, Myocardial infarction, Angiogenesis, Apoptosis

## Abstract

**Background:**

The Chinese medicine Huoxue Wentong Formula (HXWTF) was used to treat thoracic obstruction and angina pectoris in clinic, which has not been investigated in myocardial ischemia-induced apoptosis and angiogenic function. Here we aimed to investigate the roles of HXWTF in rats with myocardial ischemia-induced apoptosis and angiogenesis disorders, as well as to reveal the potential mechanisms.

**Methods:**

Male SD rats were subjected to coronary artery ligation followed by HXWTF (420, 840 and 1680 mg/kg/day, p.o.) or isosorbide mononitrate (6.3 mg/kg/day, p.o.) treatment for 4 weeks. Electrocardiogram (ECG) and Echocardiography (ECHO) were used to measure cardiac function. Hematoxylin and eosin (H&E) staining and CD34/α-SMA immunohistochemical staining were performed to observe the ischemic heart sections pathological changes and angiogenesis. Then, the effects on cardiomyocyte apoptosis of H9c2 and tube formation of HCMECs were observed, as well as the changes in the levels of total calmodulin dependent protein kinase II (t-CaMKII), phosphorylated CaMKII (p-CaMKII), oxidized CaMKII (ox-CaMKII), CD34, and Bcl-2/Bax ratio were detected.

**Results:**

Rats with coronary artery ligation exhibited abnormal cardiac function, enlarged myocardial space, disorderly arranged myocardial fibers, inflammatory cells infiltrated, and aggravated myocardial cell apoptosis, along with angiogenesis dysfunction. The expressions of CD34, p-CaMKII, and ox-CaMKII were elevated and Bcl-2/Bax ratio was diminished in ischemic hearts and H/SD-treated H9c2 or HCMECs, while HXWTF treatment completely rescued angiogenic dysfunction, inhibited cardiomyocyte apoptosis, and down-regulated cardiac CaMKII oxidation and phosphorylation activities.

**Conclusion:**

Our study demonstrates that HXWTF improves myocardial infarction possibly through inhibiting CaMKII oxidation and phosphorylation levels, facilitating angiogenic function and alleviating cardiomyocyte apoptosis. Thus, therapeutics targeting CaMKII activities may be a promising strategy for rescuing ischemic cardiomyopathy.

## Background

Myocardial infarction (MI) is a serious threat to human life and health, and its morbidity and mortality have ranked first among all kinds of cardiovascular diseases. MI is a syndrome of myocardial cell necrosis, which is caused by severe and persistent ischemia and hypoxia of the local myocardium induced by coronary artery occlusion [1, 2]. It is well known that myocardial ischemia exacerbates the pathological damage of MI through impairing ischemic vascular remodeling and collateral formation [3, 4]. Therefore, inhibition of myocardial apoptosis and promotion of angiogenesis are of great importance for the treatment of ischemic heart disease such as MI. Although the current therapeutic strategy of MI has greatly improved the clinical symptoms and reduced the mortality of patients, the rates of coronary restenosis and graft failure, along with vascular occlusion is still relatively high, they fail to repair the necrotic heart tissues, and avoid the occurrence of myocardial remodeling after MI, which seriously affects the prognosis of MI [5, 6]. Therefore, oral drugs and other non-invasive therapies are still the first choice and complementary invasive therapies to ameliorate blood flow of ischemic heart tissues.

Calmodulin dependent protein kinase II (CaMKII) is a multifunctional serine/threonine protein kinase and is one of the largest protein kinase families in organism. Some studies have shown that myocardial ischemia–reperfusion injury and MI can activate CaMKII via oxidation and phosphorylation, which resulting in myocardial fibrosis, inflammation, apoptosis and necrosis, and eventually leading to heart failure and arrhythmia [3, 7, 8]. In addition, CaMKII plays a key role in maintaining myocardial structure and function, as well as in cardiac contraction [9, 10]. Therefore, down-regulation of CaMKII activity is an important target for the treatment of ischemic heart disease.

Huoxue Wentong Formula (HXWTF) originated from Danshen Yin and has been modified appropriately, which has the effects of activating blood circulation and removing blood stasis, invigorating qi and relieving pain. In the previous small sample clinical study, we observed that the symptoms of myocardial ischemia significantly improved after treatment with HXWTF for 4 weeks. However, the specific therapeutic mechanism of HXWTF remains elusive. Thus, the purpose of the present study was to evaluate the angiogenic function and anti-apoptotic effect of HXWTF in the ischemic heart and hypoxia/serum deprivation (H/SD)-treated human cardiac microvascular endothelial cells (HCMECs) or H9c2 cells and to reveal HXWTF promoting cardiac angiogenesis and inhibiting apoptosis are related to CaMKII oxidation and phosphorylation activities.

## Methods

### Animals and surgery

All the animal experiments were approved by the Institutional Animal Care and Use Committee of Guang’anmen Hospital, China Academy of Chinese Medical Sciences (IACUC-GAMH-2019-005). Ten to twelve-week-old male Sprague–Dawley (SD) rats were purchased from Beijing HFK Bioscience Co., Ltd (Beijing, China) and reared in a standard experimental animal laboratory with free access to food and water. Rats were anesthetized and subjected to ischemia by the left anterior descending (LAD) coronary artery ligation as previous reported [11, 12]. Ischemic heart tissues were evaluated by electrocardiography (ECG). After operation, rats were randomly divided into six groups: (1) Sham group (without coronary artery ligation); (2) Model group (coronary artery ligation); (3) Positive group (model rats treated with 6.3 mg/kg isosorbide mononitrate sustained-release tablets); (4–6) HXWTF1, HXWTF2, and HXWTF3 groups (model rats treated with HXWTF 420, 840 or 1680 mg/kg, respectively). HXWTF and isosorbide mononitrate sustained release tablets (AstraZeneca) were dissolved in distilled water and given intragastric administration, respectively. The dosage of intragastric administration was the equivalent dose converted by the surface area of the human. The sham group and model group were given equivalent distilled water. Isosorbide mononitrate sustained release tablets and HXWTF were intragastrically administrated once daily after operation for consecutive 4 weeks.

### Preparation of HXWTF

HXWTF contains the following ingredients: *Salvia miltiorrhiza * Bunge 30 g, *Cinnamomum cassia * (L.) 10 g, *Ligusticum striatum * DC. 9 g, *Caulis spatholobi* 10 g, *Paeonia lactiflora * Pall. 10 g, *Codonopsis pilosula * (Franch.) 10 g. HXWTF is a ten times concentrated granule made from six Chinese medicinal herbs mentioned above. The concentrated granules of single traditional Chinese medicine were prepared by five steps including water boiling extraction, low temperature concentration, spray drying, dry granulation and packaging. The batch numbers of the above six Chinese herbal formula granules are 18080058, 18060154, 18090064, 18050094, 18040048, 18060048, respectively. Because most of the active ingredients of traditional Chinese medicine are mostly unclear, the thin-layer chromatography (TLC) is used to qualitatively identify the dispensing granules of traditional Chinese medicine. The TLC identification results of formula granules achieved the standard of Chinese Pharmacopoeia (version 2010). An additional PDF file shows the quality control results of HXWTF in more detail (see Additional file 1). All herbal materials, dispensing granules, and quality control data of HXWTF were supplied by Sichuan Neo-Green Pharmaceutical Technology Development Co., Ltd (Sichuan, China).

### Electrocardiogram (ECG)

At the end of the myocardial ischemia operation period, rats were anaesthetized, and the ECG was monitored using RM-6240 Multichannel Biological Signal Acquisition and Processing System (Chengdu Instrument Factory Manufacturing, Chengdu, China). The subcutaneous platinum electrodes were placed in lead II arrangement. Body temperature was maintained at 37 °C. The ECG samples of each rat for 3 min were analyzed.

### Echocardiography (ECHO)

Cardiac left ventricular geometry and contractile function were measured after 2 weeks of HXWTF treatment in rats with MI, rats were anaesthetized with ventilation by inhalation of isoflurane. M-mode echocardiography was obtained with a high-resolution imaging system (VisualSonics Vevo 2100, Toronto, Canada) equipped with a frequency transducer (frequency: 30 MHz). Ejection fraction (EF/%) and fractional shortening (FS/%) were measured and calculated using Vevo LAB software (version 3.1.1). To minimize the influences of different heart rates and body temperature on left ventricular function, the flow of isoflurane was adjusted to anaesthetize the rat, while the heart rates was maintaining at 400–500 beats per minute and the body temperature was maintained at 37 °C by a heating pad.

### Evans blue (EB) and 2,3,5-triphenyltetrazolium chloride (TTC) double staining

To observe the myocardial infarct size, the ligature around the coronary artery was reoccluded and 1 mL of 0.5% Evans blue (Solarbio, Beijing, China) was injected into the left ventricular cavity after ischemic injury. The heart was quickly removed, frozen at − 20 °C and then sliced horizontally to yield fives slices. Heart slices were incubated in PBS phosphate buffer containing 2% TTC solution (Solarbio) at 37 °C for 20 min, and then slices were fixed in 4% paraformaldehyde. Images were acquired with a digital camera.

### TUNEL staining

Myocardial apoptosis in heart tissues was detected by the situ TUNEL apoptosis detection kit (Roche, Basel, Switzerland), according to the manufacturer’s protocols. Briefly, the formalin-fixed paraffin embedded tissues sections were incubated with proteinase K for 15 min and washed three times with PBS. The sections were incubated with TdT-enzyme TUNEL reaction mixture at 37 °C for 1 h, and then conjugated with antibody labeled with horseradish peroxidase (HRP) for 30 min. The interaction of antigen and antibody was observed by DAB. And then, the sections were then counterstained with hematoxylin. Samples were visualized using Pannoramic Digital Slide Scanners (3DHISTECH, Pannoramic 250 Flash III, Hungary). To assess myocardial apoptosis, the average count of six fields in each slide was calculated, and the number of apoptotic cardiomyocytes in TUNEL positive staining cells in each group were analyzed with Image-Pro Plus 5.1.

### Histological analysis

To evaluate the cardiac architecture and pathological changes in heart tissues. Formaldehyde-fixed heart tissues specimens were embedded in paraffin, and 5 μm sections were prepared and stained with hematoxylin and eosin (H&E), immune-stained with anti-CD34 (Abcam, Cambridge, MA) and anti-α-SMA antibody (Abcam) according to standard operation procedures. Images were acquired with Pannoramic Digital Slide Scanners. To assess revascularization in the heart, the average count of five fields in each sample was determined, and the area of vessels in staining samples were analyzed with Image-Pro Plus 5.1.

### Cell culture

Rat myocardial cells (H9c2) and Human cardiac microvascular endothelial cells (HCMECs) were purchased from Jennio-bio (Guangzhou, China). H9c2 were cultured in Dulbecco’s modified Eagle’s medium (DMEM, Gibco, USA) supplemented with 10% fetal bovine serum (Gibco, Australia) and 1% streptomycin/penicillin (Solarbio), and HCMECs were cultured in Endothelial Cell Medium (ECM, ScienCell, USA) supplemented with 10% fetal bovine serum and 1% streptomycin/penicillin in the 5% CO_2_ incubator (Thermo Fisher Scientific, Waltham, MA, USA) at 37 °C for 48 h.

Cultured rats H9c2 and HCMEC were stimulated with hypoxia/serum deprivation (H/SD) to mimic in vivo myocardial ischemic injury as previously described [13]. Briefly, the cells were washed with PBS, and cultured in Hanks buffer (Gibco, Grand Island, NY, USA). After that, the cells were incubated in an anoxic chamber (95% N_2_, 5% CO_2_) (Thermo Fisher Scientific) at 37 °C for 4 h, and added serum containing HXWTF to the culture as H/SD simultaneously. In the control group, cells were maintained at normoxia (95% O_2_, 5% CO_2_) for equivalent periods of time. We used cultured cells from 2 to 6 passages for this experiment. H9c2 and HCMECs dishes were randomly divided into four groups: (1) Con (Normoxia group); (2) H/SD (Hypoxia/serum deprivation group); (3) H/SD + 5 μmol/L KN-93 [(Merck Millipore, Billerica, MA, USA), H/SD plus KN-93 group], KN-93 is an inhibitor of CaMKII; (4) H/SD + HXWTF (H/SD plus serum containing HXWTF group). We used HXWTF2 group rats to prepare drug serum. Blood was taken from abdominal aorta, and then, centrifuged at 3500 rpm for 15 min, and the supernatant was retained. After inactivation by heating at 56 °C for 30 min and filtration sterilization by 0.22 μm Millipore filter, the serum containing HXWTF was stored in a refrigerator at − 80 °C. The serum containing HXWTF with a final concentration of 10% was used in the experiment.

### Caspase 3/8 activity assay

Caspase 3/8 activity in H9c2 cells were measured by Elisa kits (Solarbio) according to the manufacturer’s instructions. The absorbance was determined by Molecular Devices SpectraMax M2 Microplate Reader (Molecular Devices, USA).

### Hoechst staining

Intracellular apoptosis was detected by using Hoechst Staining Kit (Beyotime Biotechnology, Shanghai, China). The treatment H9c2 cells were washed with PBS and fixed in 4% paraformaldehyde for 10 min at room temperature. After fixation, the cells were washed three times with PBS and incubated in Hoechst staining solution for 5 min at room temperature. Then, cells were washed again with PBS and the fluorescence (at 350 nm excitation and 460 nm emission) was determined by a Leica laser scanning confocal microscope (Leica TCS SP5 II, Leica Microsystems).

### Angiogenesis assay

To assess angiogenic capability in vitro. HCMECs seeded on 2 × 10^4^ cells per well were incubated with ECM medium in 96-well plates precoated with Matrigel (BD, Bedford, MA, USA) and incubated at 37 °C with 5% CO_2_ for 2 h to form capillary-like tubes. Representative images of tubes in each well were obtained by a microscope (Leica TCS SP2 Microsystems, Heidelberg, Germany).

### Immunofluorescent staining

The hypoxic HCMECs were stained with CD34 immunofluorescence. After the ECM medium was harvested, HCMECs were fixed in 4% paraformaldehyde for 20 min at room temperature. After fixation, the cells were washed three times with PBS for 5 min. Next, added to 0.1% Triton X-100 citrate solution for 30 min, and then washed three times with PBS, and then blocked with 5% BSA for 60 min. The cells were incubated overnight at 4 °C with rabbit polyclonal anti-CD34 (1:200) (Abcam). The cells were then incubated for 60 min at room temperature with Alexa Fluor 488-Conjugated AffiniPure goat anti-mouse/rabbit IgG (1:500), After incubation, the cells were washed three times with PBS for 5 min and nuclei were counterstained with DAPI (1:1000), and then washed three times with PBS for 5 min and were visualized via a STED Confocal Microscope (TCS SP8 STED, Leica, Germany).

### Immunoblot analysis

Proteins were extracted from heart tissues and cell samples with RIPA buffer containing cocktail (Sigma Aldrich), and protein qualification was performed using a BCA Protein Assay Kit (Thermo Fisher Scientific). Lysates were separated on 10% or 12% SDS-PAGE and transferred to a PVDF membrane (Millipore). The membranes were blocked in tris-buffered saline with 5% nonfat milk or 5% bovine serum albumin (BSA) for 1 h, and then incubated with primary antibody overnight at 4 °C, followed by incubation with the horseradish peroxidase (HRP)-conjugated secondary antibodies (Santa Cruz) for 1 h at room temperature after standard washing procedures. The primary antibodies against CD34 (Abcam), CaMKII (GeneTex, CA, USA), ox-CaMKII (GeneTex), p-CaMKII (Thr286) (Thermo Scientific), Bcl-2 (CST), Bax (CST), β-actin (ZSGB-BIO). The signals were detected by chemiluminescence FluorChem™FC3 system (Protein Simple, USA). Relative luminescence intensity was analyzed by Quantity One software (Bio-Rad Laboratories, Hercules, CA, USA).

### Statistical analysis

All data are presented as mean ± standard deviation (SD). The multi-group comparisons were determined by one-way analysis of variance followed by least significant difference (LSD) post hoc multiple comparisons test and the two group comparisons were performed using Student *t* test of variance. All analyses were performed with statistical software SPSS 16.0 (SPSS Inc., Chicago, IL, USA). Differences were considered statistically significant at *p* < 0.05.

## Results

### HXWTF improved the abnormal cardiac function in MI

**Fig. 1:**
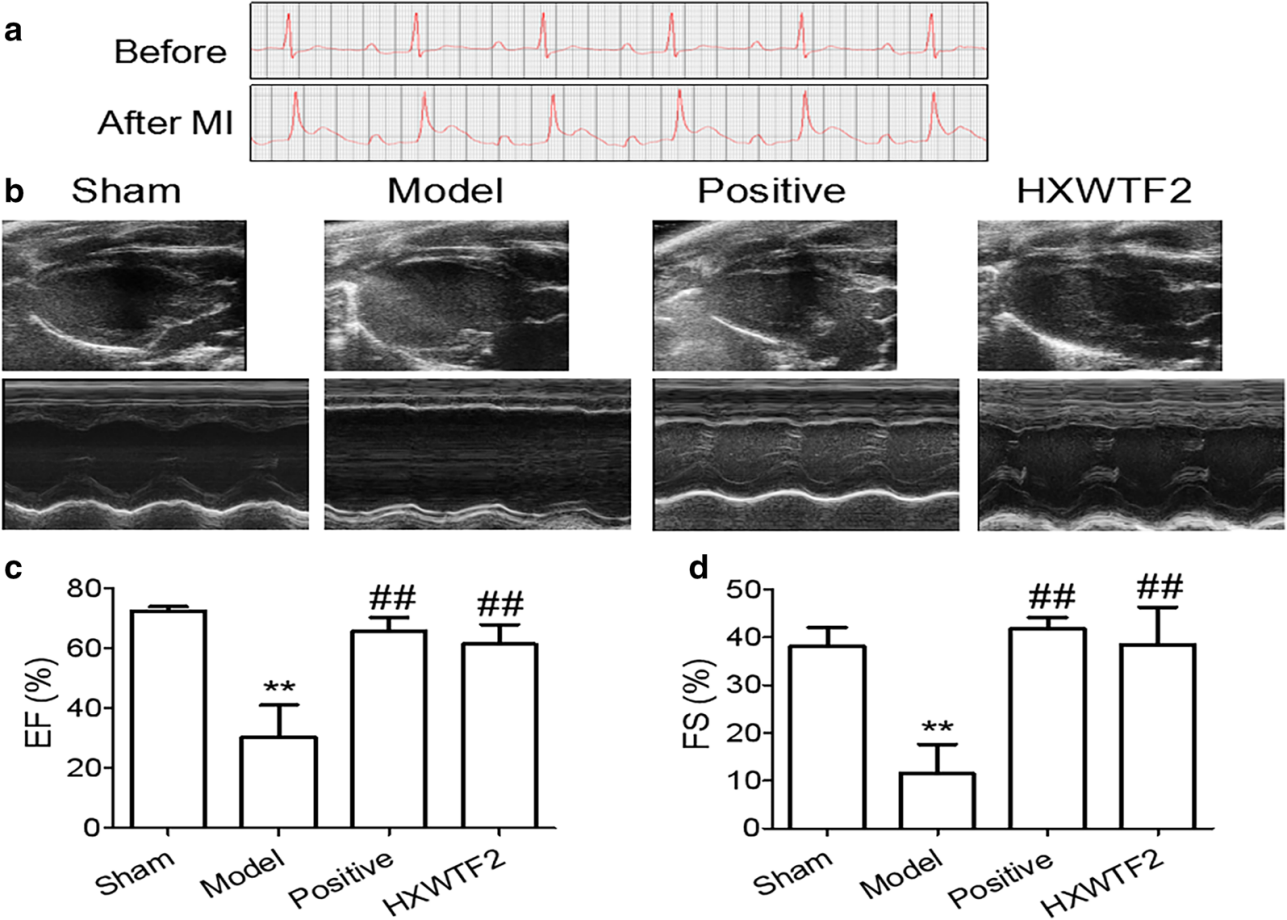
HXWTF restored cardiac function after myocardial infarction (MI) in rats. Representative typical surface electrocardiogram recordings (**a**) and representative M-mode echocardiographic images (**b**) in rats with myocardial ischemia. Ejection fraction (EF) values (**c**). Fractional shortening (FS) values (**d**) Sham, Sprague–Dawley (SD) rats without coronary artery ligation; Model, SD rats with coronary artery ligation; Positive, 6.3 mg/kg isosorbide mononitrate sustained release tablets-treated model rats; HXWTF1, 420 mg/kg Huoxue Wentong Formula (HXWTF)-treated model rats; HXWTF2, 840 mg/kg HXWTF-treated model rats; HXWTF3, 1680 mg/kg HXWTF-treated model rats. Values are presented as mean ± SD (n = 8 per group)

In order to evaluate the success of MI model, the ECG of each model rat was measured. The results showed that the ST segment elevated significantly after MI compared with before MI (Fig. 1a). In this experiment, the success rate of MI model was more over 90%, and the mortality rate was less than 5%. ECHO was used to detect cardiac function after MI. As shown in Fig. 1b–d, Ejection Fraction (EF) and Fractional Shortening (FS) were decreased markedly compared with the sham group. However, HXWTF treatment promoted the functional recovery of post-ischemic hearts, as confirmed by increased EF and FS compared with the model group, and isosorbide mononitrate had similar effects on the recovery of ischemic cardiac function as well (Fig. 1b–d). These data demonstrated that MI rats developed the characteristics of cardiac dysfunction, and HXWTF had the effects of improving the cardiac function after MI.

### HXWTF reversed aggravation of myocardial ischemic injury

**Fig. 2:**
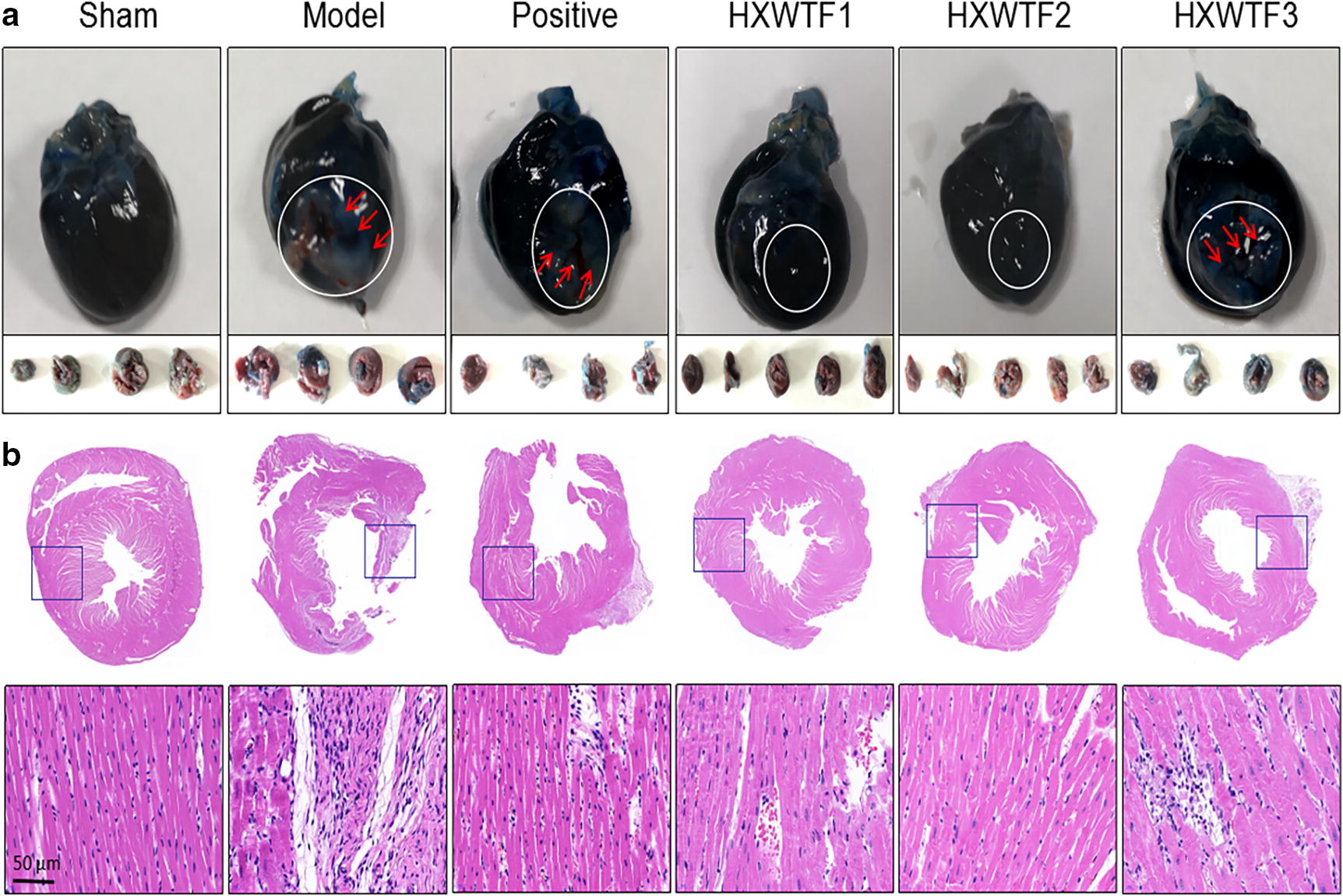
HXWTF attenuated myocardial ischemic injury in model rats. Representative photos of heart sections stained with EB/TTC, four to five heart images at the bottom represent different transverse sections of the upper heart (**a**) and H&E (**b**). Scaling is as indicated, Scale bar 50 μm. Red arrows and white circles indicate myocardial infarction area in heart tissues. n = 8 per group

Next, we evaluated post-ischemic MI and cardiac structural changes. EB/TTC double staining and H&E staining indicated that the ischemic heart suffered from serious pathological injury, including increased infarct size, enlarged ischemic myocardial space, disorderly arranged myocardial fibers, and many inflammatory cells infiltrated (Fig. 2a, b). However, compared with the model group, HXWTF and isosorbide mononitrate treatment could shrink infarct size, maintain the integrity of myocardial fibers structure and inhibit inflammatory cells infiltration (Fig. 2a, b). These results indicated that HXWTF improved the pathological changes of MI.

### HXWTF relieved cardiomyocyte apoptosis and restored the Bcl-2/Bax ratio

**Fig. 3:**
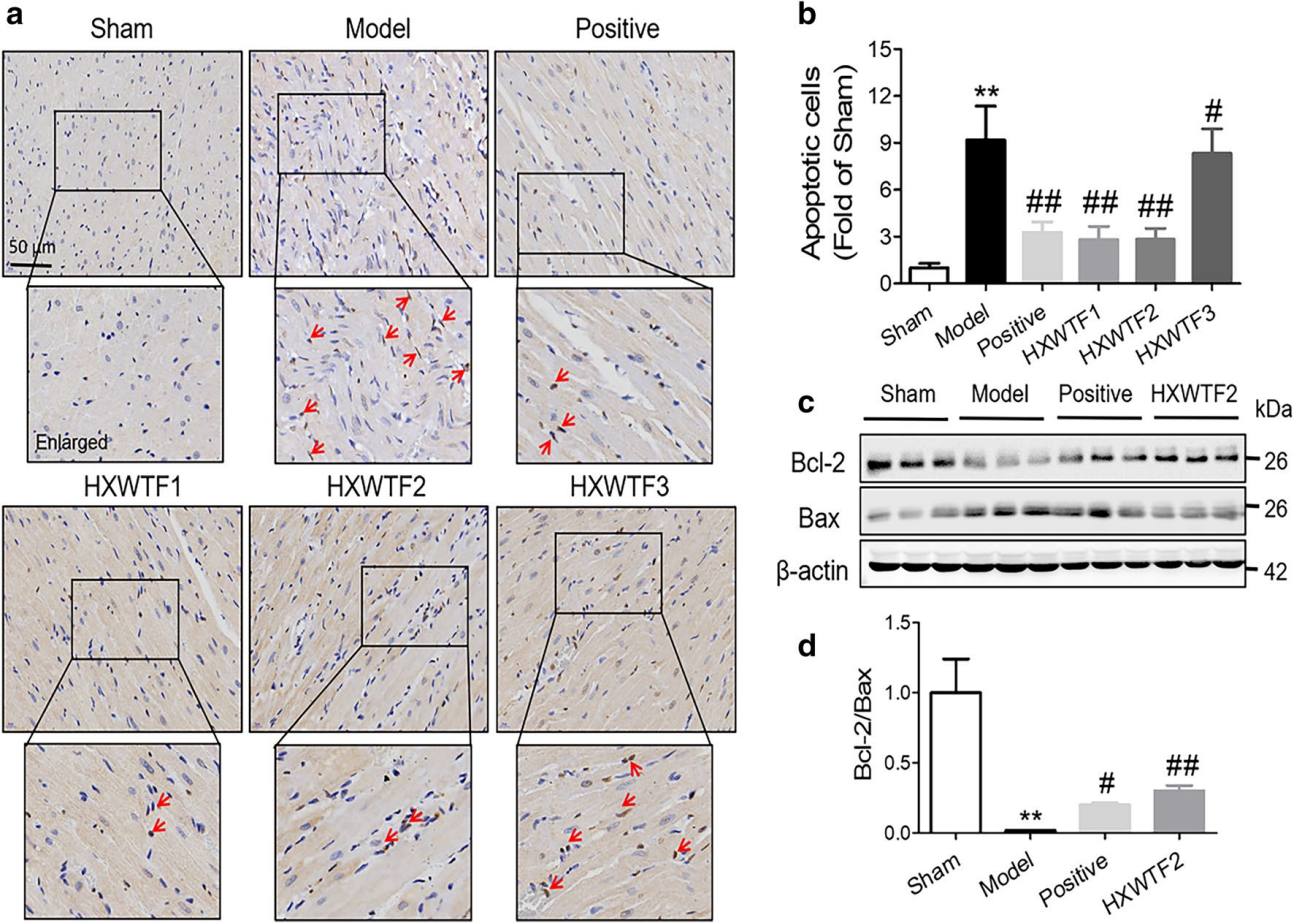
HXWTF inhibited cardiomyocyte apoptosis of post-ischemic hearts. Myocardial apoptosis was analyzed by TUNEL staining (**a**). Red arrow indicates apoptotic cardiomyocytes in heart tissues. Scaling is as indicated, Scale bar 50 μm. Quantificative analysis of the number of apoptotic cardiomyocytes (**b**). Representative western blot bands were shown for Bcl-2 and Bax in heart tissues (**c**). Quantitative analysis of Bcl-2 and Bax expressions, as well as Bcl-2/Bax ratio (**d**). The results were normalized to the sham group. **P< 0.01, *P< 0.05 compared to the sham group; ^##^P< 0.01, ^#^P< 0.05 compared to the model group. Values are presented as mean ± SD (n = 4 per group)

Cardiac ischemia is known to cause an increase in apoptotic cardiomyocytes, which leading to heart dysfunction. Hence, we used TUNEL staining and Bcl-2/Bax to assess the myocyte apoptosis after MI. Our results demonstrated that the number of apoptotic cardiomyocytes increased and the Bcl-2/Bax ratio reduced greatly in model group compared with the sham group, whereas HXWTF and isosorbide mononitrate treatment effectively inhibited ischemia-induced up-regulation of the number of apoptotic cardiomyocytes and restored Bcl-2/Bax ratio (Fig. 3a–d). Collectively, HXWTF had obvious anti-apoptotic effect on myocardium.

### HXWTF promoted cardiac vessels generation after myocardial ischemia

**Fig. 4:**
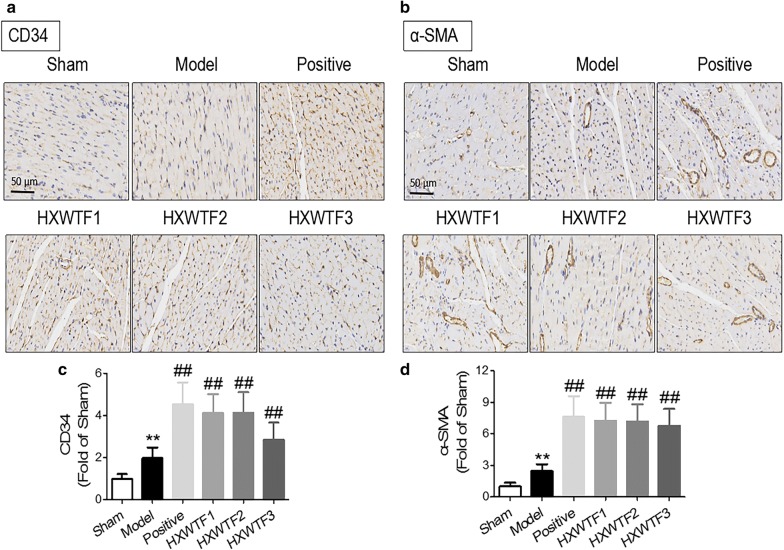
HXWTF promoted cardiac vessels generation in rats with MI. Representative micrographs of heart sections with immunohistochemical staining of CD34 and α-SMA (**a**, **b**). Quantitative analysis of CD34-positive cells areas and α-SMA-positive capillary areas were performed in heart tissues from each group, respectively (**c**, **d**). Scaling is as indicated, Scale bar 50 μm. Values are presented as mean ± SD (n = 8 per group)

**Fig. 5:**
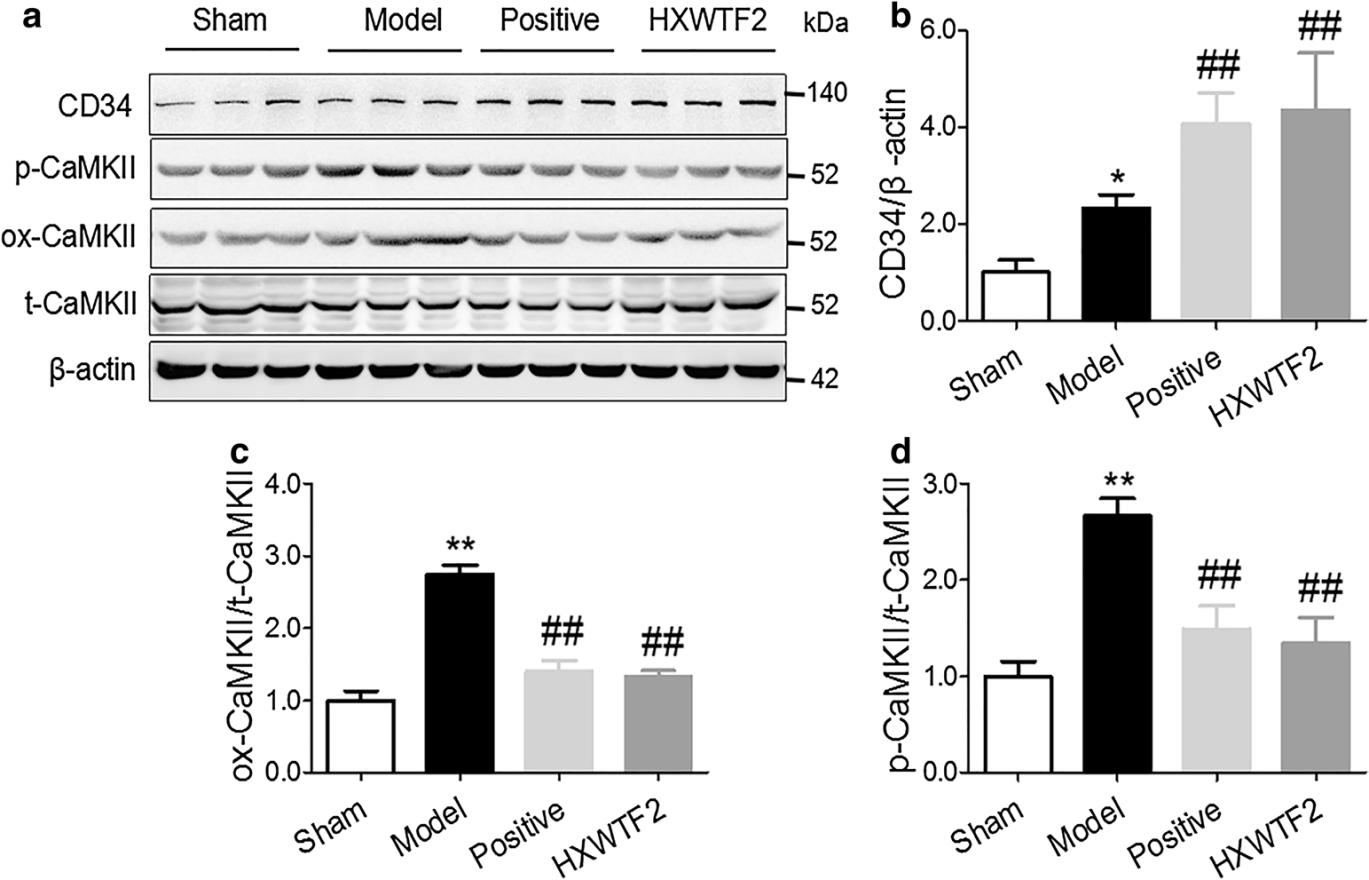
HXWTF inhibited the levels of p-CaMKII, ox-CaMKII, and elevated the CD34 expression. Representative western blot bands were shown for CD34, ox-CaMKII, p-CaMKII on Thr286, total-CaMKII and β-actin in heart tissues (**a**). Quantitative analysis of CD34, ox-CaMKII, p-CaMKII on Thr286 and total-CaMKII expressions (**b**–**d**). The results were normalized to the sham group. CaMKII, calmodulin (CaM) dependent protein kinase II; ox-CaMKII, oxidized CaMKII; p-CaMKII, phosphorylated CaMKII. **P< 0.01, *P< 0.05 compared to the sham group; ^##^P< 0.01, ^#^P< 0.05 compared to the model group. Values are presented as mean ± SD (n = 4 in each group)

As we all know, collateral vessel formation is seriously damaged in patients with MI, which will lead to myocardial tissues more susceptible to critical ischemia. CD34/α-SMA immunohistochemical staining and western blotting were used to assess positive vessels generation at 4 weeks post-operation. The results showed that CD34 and α-SMA expressions were associated with a significant increase in the model group compared with sham rats, this increase was most likely caused by local ischemia stimulation. However, compared with the model group, the expressions of CD34 and α-SMA were markedly elevated in the HXWTF and isosorbide mononitrate treatment groups (Figs. 4a–d; 5a, b), suggesting that HXWTF promotes vessels generation in ischemic heart.

### HXWTF inhibited p-CaMKII and ox-CaMKII expressions and elevated the CD34 level of ischemic heart

In addition, to understand the target proteins for angiogenesis dysfunction. We measured the expressions of related proteins in the ischemic heart tissues. The data presented that CaMKII oxidation and phosphorylation levels were markedly elevated, CD34 expression was increased slightly in the model group compared with the sham group (Fig. 5a–d). Meanwhile, the expression of CD34 was further significantly enhanced (Fig. 5a, b), and the levels of CaMKII oxidation and phosphorylation were dramatically reduced in the HXWTF and isosorbide mononitrate treatment (Fig. 5c, d). Together these results suggested that down-regulation of CaMKII oxidation and phosphorylation activities may play a critical role in revascularization of MI.

### HXWTF attenuated cardiomyocyte apoptosis and down-regulated the expressions of p-CaMKII and ox-CaMKII in H/SD-treated H9c2

**Fig. 6:**
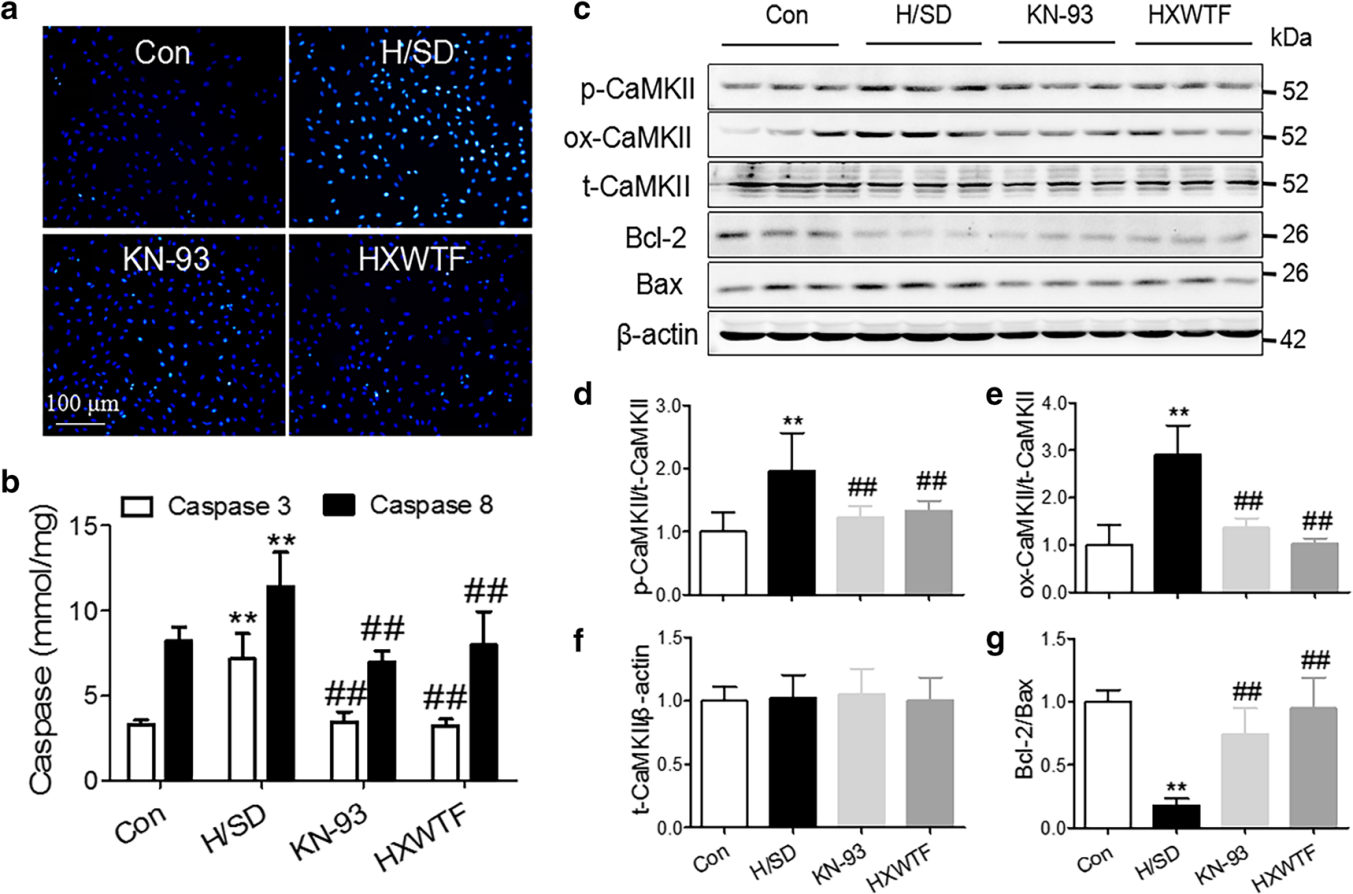
LMWF alleviated cardiomyocyte apoptosis in H/SD-treated H9c2. Hoechst staining for H9c2 treated with H/SD (**a**). Scaling is as indicated, Scale bar 100 μm. Caspase 3 and Caspase 8 activities in H/SD-treated H9c2 were detected by Elisa (**b**). Western blot analysis of p-CaMKII, ox-CaMKII, and total CaMKII expressions, along with Bcl-2/Bax ratio in H/SD-treated H9c2 (**c**–**g**). Con, Normoxia group; H/SD, hypoxia/serum deprivation group. **P< 0.01, *P< 0.05 compared to the sham group; ^##^P< 0.01, ^#^P< 0.05 compared to the model group. Values are presented as mean ± SD (n = 4 per group)

To further confirm if HXWTF can inhibit H9c2 cardiomyocyte apoptosis under hypoxia/serum deprivation (H/SD) condition, we administered the rats of serum containing HXWTF into H9c2 for 4 h intervention. Hoechst staining, caspase 3/8 activity and Bcl-2/Bax ratio were used to evaluate cardiomyocyte apoptosis. The results indicated that H/SD induced cardiomyocyte apoptosis, the nuclei were densely stained or fragmented densely stained with white color and the level of caspase 3/8 greatly increased. Conversely, the number of deep stained nuclei in the serum containing HXWTF and KN-93 (an inhibitor of CaMKII) were significantly reduced, and most of the nuclei were normal blue, and caspase 3/8 activity decreased significantly compared with the H/SD group (Fig. 6a, b). Moreover, the results of western blotting showed that p-CaMKII and ox-CaMKII levels increased significantly, Bcl-2/Bax ratio decreased markedly of H9c2 treated with H/SD (Fig. 6c–g). However, KN-93 and HXWTF treatment significantly reduced H/SD-elevated caspase 3/8 and CaMKII activities, restored the Bcl-2/Bax ratio (Fig. 6b–g). These results further revealed that HXWTF alleviated cardiomyocyte apoptosis via elevating Bcl-2/Bax ratio and inhibiting CaMKII activities in H/SD treated H9c2. Likewise, in consistent with the results revealed in rats with MI.

### HXWTF promoted tube formation and decreased the expressions of p-CaMKII and ox-CaMKII in H/SD-treated HCMECs

**Fig. 7:**
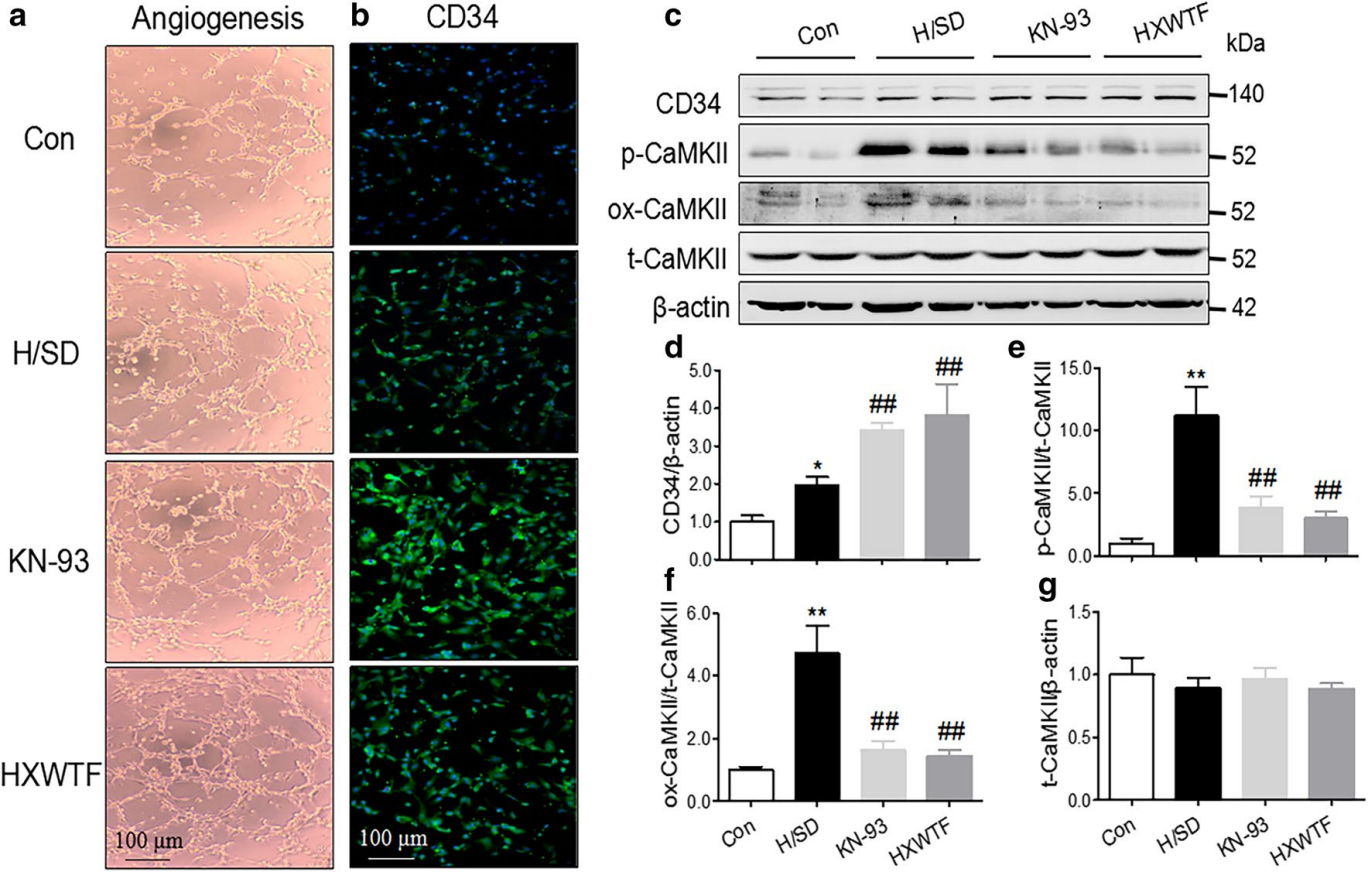
HXWTF improved angiogenic function and blocked CaMKII oxidation and phosphorylation of HCMECs treated with H/SD. Tube formation on Matrigel was examined in HCMECs (**a**). Immunofluorescence staining of HCMECs were positive for CD34 (**b**). Scaling is as indicated, Scale bar 100 μm. Western blot analysis of CD34, p-CaMKII, ox-CaMKII and total CaMKII expressions in H/SD-treated HCMECs (**c**–**g**). HCMECs, Human cardiac microvascular endothelial cells. **P< 0.01, *P< 0.05 compared to the sham group; ^##^P< 0.01, ^#^P< 0.05 compared to the model group. Values are presented as mean ± SD (n = 4 per group)

Furthermore, in order to observe if HXWTF can ameliorate endothelial cells angiogenesis obstacle under H/SD condition, we administered the rats of serum containing HXWTF into HCMECs. Tube formation assay, CD34 immunofluorescence staining, and western blotting were used to determine cardiac microvascular endothelial cells angiogenesis. These results indicated that H/SD increased tube formation and CD34 expression of HCMECs, significantly enhanced p-CaMKII and ox-CaMKII levels of HCMECs treated with H/SD compared with control group (Fig. 7a–d). However, HXWTF or KN-93 further augmented tube formation and CD34 expression, which accompanied by the decrease of p-CaMKII and ox-CaMKII levels (Fig. 7a–g). These data are consistent with those in vivo, indicating that HXWTF-mediated angiogenesis possibly via inhibiting CaMKII oxidation and phosphorylation.

## Discussion

The number of patients with MI is increasing gradually, which deteriorates the quality of life for patients worldwide and has become one of the cardiovascular diseases with the highest mortality [14]. In the current study, our results revealed that rats with MI exhibited abnormal cardiac function, increased infarct size, ischemic pathological changes, led to apoptosis of cardiac myocytes and vascular regeneration dysfunction. However, HXWTF treatment restored heart function, reduced the myocardium infarct size, inhibited myocardial apoptosis, and improved angiogenic function through blocking CaMKII phosphorylation at Thr286 and CaMKII oxidation under ischemic and hypoxic conditions. Therefore, these data indicated that HXWTF improves myocardial ischemia in part through inhibiting CaMKII phosphorylation and oxidation promoting angiogenesis and retarding apoptosis.

Myocardial ischemia prevents the necessary blood flow to the heart tissues, leading to coronary angiogenesis obstruction [15]. Cardiac neovascularization is an important protective mechanism of ischemic heart disease. Recent studies have revealed that the hearts of neonatal mice use a new mechanism to establish collateral arteries to cope with injury. Arterial endothelial cells migrated from artery to collateral artery along existing capillaries to achieve neonatal cardiac regeneration, which may be used as a potential therapy for ischemic heart disease [6]. Moreover, previous studies have confirmed that bone marrow-derived endothelial progenitor cells (EPCs) had the potential to differentiate into endothelial cells and injected EPCs into the hearts of MI can improve cardiac function via promoting neovascularization [16–18]. Thus, EPCs play an important role in protecting vascular endothelium and the formation of new blood vessels in cardiovascular disease. However, the specific mechanisms are still largely complicated. Here, our findings showed that HXWTF ameliorates myocardial damage through promoting angiogenesis in ischemic heart and tube formation in hypoxic endothelial cells by inhibiting CaMKII activation, which may provide theoretical basis and therapeutic mechanism for the treatment of MI.

Apoptosis is a common phenomenon in physiological processes, but excessive cell apoptosis will directly lead to the decline of tissue and organ function. The increase and acceleration of cardiomyocyte apoptosis may be a key factor in MI. Previous studies have shown that ischemia/reperfusion injury can cause ROS accumulation and trigger caspase 3 activation leading to cardiomyocyte apoptosis in ischemic heart [19–21]. Apoptosis is characterized by chromatin condensation, DNA fragmentation and protein cleavage [22, 23]. There are two major pathways mediate activating endotheliocyte apoptosis. Interaction with death receptor ligand, such as Fas, result in the formation of death-induced Fas associated death domain (Fadd) and initiator caspase 8, which then directly activates caspase 3, ultimately initiates cell death (extrinsic apoptotic pathway). Furthermore, death signals cause mitochondrial activation and releasing of mitochondrial intermembrane space proteins to form the apoptosome and activates caspase 9, eventually trigger cell death (intrinsic apoptotic pathway) [24–26]. Better and deeper understanding the exact mechanisms of cardiomyocyte apoptosis may lead to important and effective therapeutic strategies that significantly diminish the incidence and mortality of ischemic heart disease, especially in MI. In this study, we have confirmed that myocardial ischemia was associated with higher number of apoptotic cardiomyocytes followed by activation of caspase 3/8 activity. Interestingly, HXWTF can markedly inhibit cardiomyocyte apoptosis by preventing the caspase 3/8 activity and enhancing Bcl-2/Bax ratio.

Calmodulin (CaM) dependent protein kinase II (CaMKII) is an important protein and has been noted for participating in the processes of several cardiovascular diseases, such as myocardial hypertrophy, myocardial apoptosis and fibrosis, inflammation, heart failure, arrhythmias, pathological cardiac remodeling, MI, myocardial ischemia–reperfusion injury and so on [27, 28]. CaMKII is a serine/threonine kinase, which exists as an enzyme complex. There are four CaMKII subtypes (α, β, γ, δ). These CaMKII subtypes are distributed in different tissues. Among them, CaMKIIδ is mainly enriched in the heart and activated by autophosphorylation or oxidation, which is implicated in heart disease [29]. Relevant evidences suggested that cardiac-specific CaMKIIδ gene deletion alleviates ischemia–reperfusion induced infarct formation, inflammatory reaction, myocardial cell death and loss of cardiac systolic function by NF-κB [9, 30]. In addition, studies have demonstrated that the absence of CaMKII after cardiac injury can attenuate cardiac remodeling and reverse the symptoms of heart failure, suggesting that CaMKII inhibition is a promising treatment for heart failure [31–33]. Moreover, CaMKII is essential for both endothelium-derived relaxation and eNOS activity in type 2 diabetic rats [34]. Meanwhile, activated CaMKII mediates apoptosis of retinal capillary endothelial cells induced by hyperglycemia via activation mitochondrial dependent and death receptor Fas/Fadd apoptosis pathways [35]. However, the effects of CaMKII activity on angiogenesis and apoptosis in ischemic heart disease has not been fully studied. In this study, we have confirmed that up-regulation of CaMKII oxidation or phosphorylation plays a critical role in ischemic-induced revascularization dysfunction and apoptosis in the heart, and then HXWTF could effectively repair the impairment of angiogenesis and inhibit myocardial apoptosis in ischemic heart and H/SD-treated HCMECs or H9c2, at least in part, may be attributable to a reduced CaMKII oxidation or phosphorylation.

## Conclusion

The present study suggests that elevation of CaMKII oxidation and phosphorylation is involved in cardiac dysfunction of MI, and HXWTF ameliorates myocardial ischemia through promoting angiogenesis and inhibiting apoptosis via down-regulation of CaMKII oxidation and phosphorylation levels. Therefore, the oxidation or phosphorylation of CaMKII may represent an important therapeutic target for the treatment of ischemic heart disease, especially MI.

## Supplementary information

**Additional file 1.** This section includes the figures of the TLC for six Chinese medicinal herbs, as well as the LC for the content determination of *Salvia miltiorrhiza* Bunge, *Ligusticum striatum * DC., and *Paeonia lactiflora * Pall.

## Data Availability

All data and materials are available from the corresponding author on reasonable request.

## Abbreviations

HXWTF: Huoxue Wentong Formula
ECG: electrocardiogram
ECHO: echocardiography
EB/TTC: Evans blue/2,3,5-triphenyltetrazolium chloride
H&E: hematoxylin and eosin
HCMECs: human cardiac microvascular endothelial cells
H/SD: hypoxia/serum deprivation
MI: myocardial infarction
CaMKII: calmodulin dependent protein kinase II
ox-CaMKII: oxidized CaMKII
p-CaMKII: phosphorylated CaMKII
TLC: thin-layer chromatography
LC: liquid chromatogram
SD: standard deviation
LSD: least significant difference
EF: ejection fraction
FS: fractional shortening
EPCs: endothelial progenitor cells
