# Supplementary material for: Huoxue Wentong Formula ameliorates myocardial infarction in rats through inhibiting CaMKII oxidation and phosphorylation
Source: Chin Med. 2020 Jan 10;15:3. doi: 10.1186/s13020-020-0285-2 (PMC6954496; doi:10.1186/s13020-020-0285-2)
Supplement: Supplementary file 1 — Additional file 1. This section includes the figures of the TLC for six Chinese medicinal herbs, as well as the LC for the content determination of Salvia miltiorrhiza Bunge, Ligusticum striatum DC., and Paeonia lactiflora Pall. [file 13020_2020_285_MOESM1_ESM.pdf]

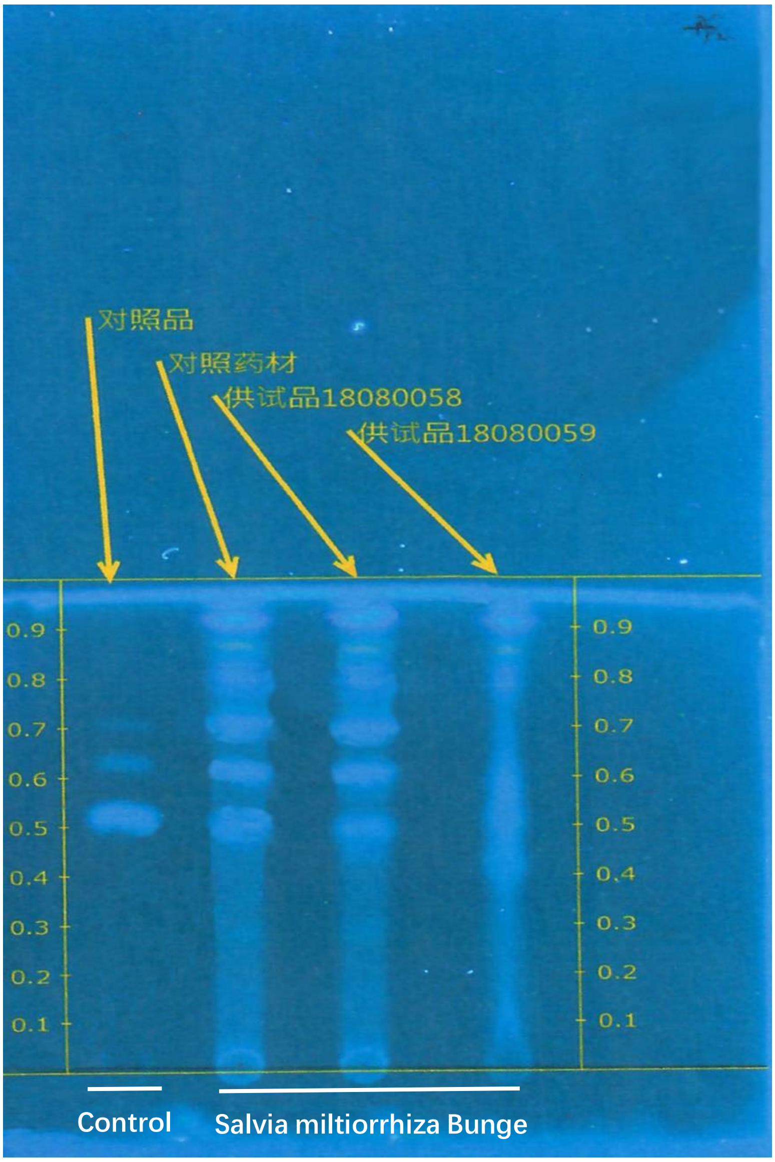

Figure. S1 The thin-layer chromatography (TLC) of *Salvia miltiorrhiza Bunge*.

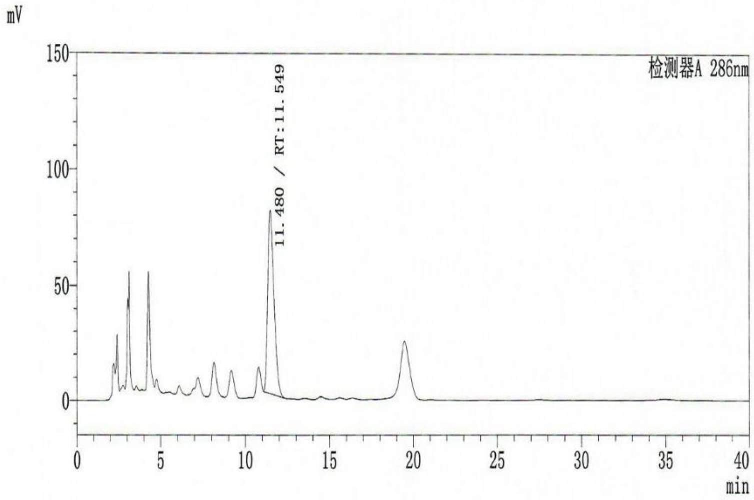

Figure. S2 The liquid chromatogram (LC) of *Salvia miltiorrhiza Bunge*.

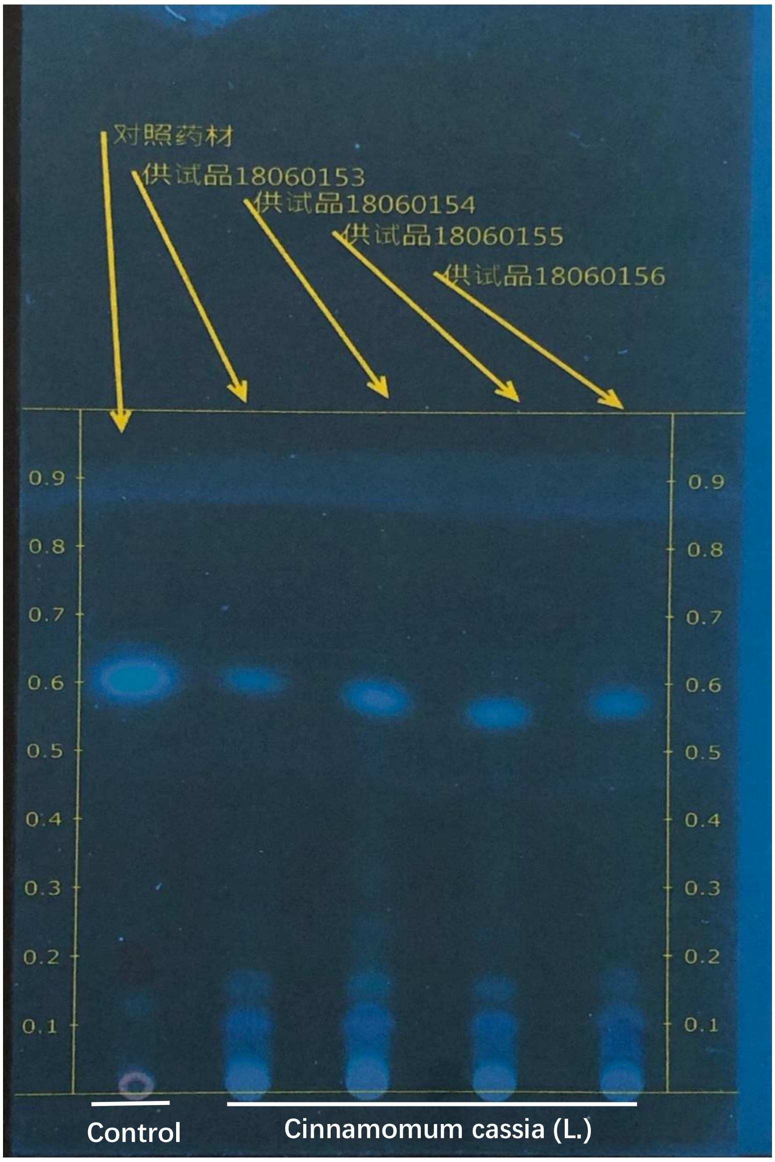

Figure. S3 The TLC of *Cinnamomum cassia (L.)*.

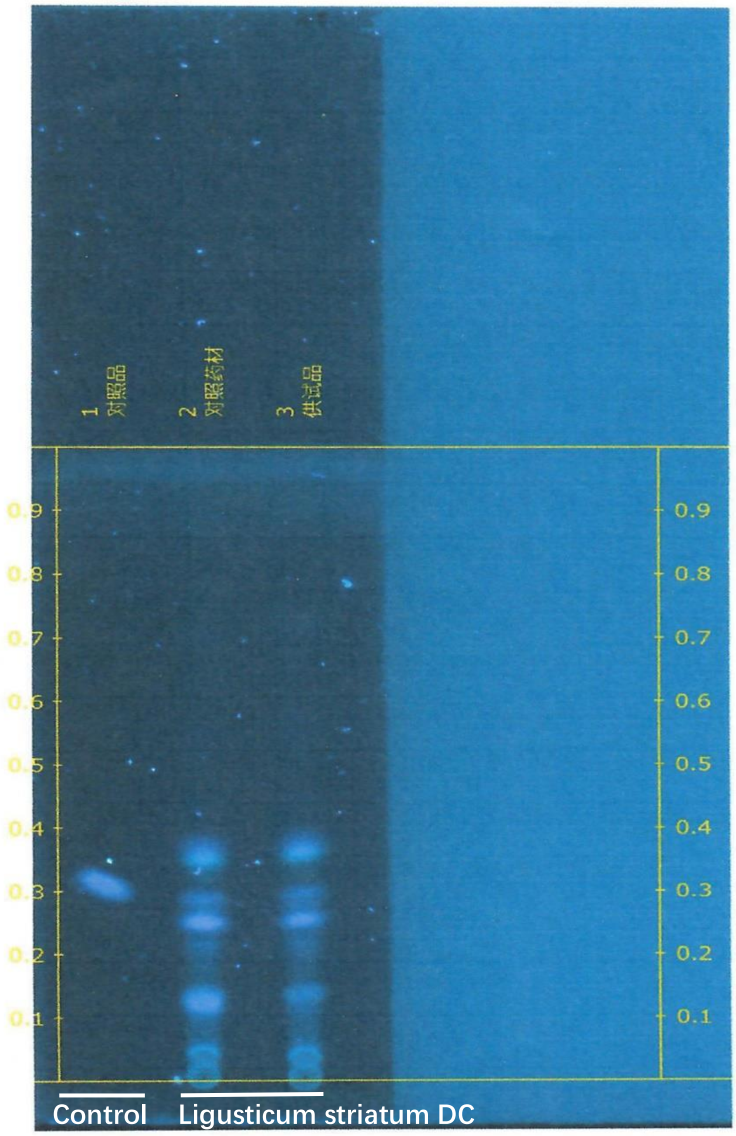

Figure. S4 The TLC of *Ligusticum striatum DC.*

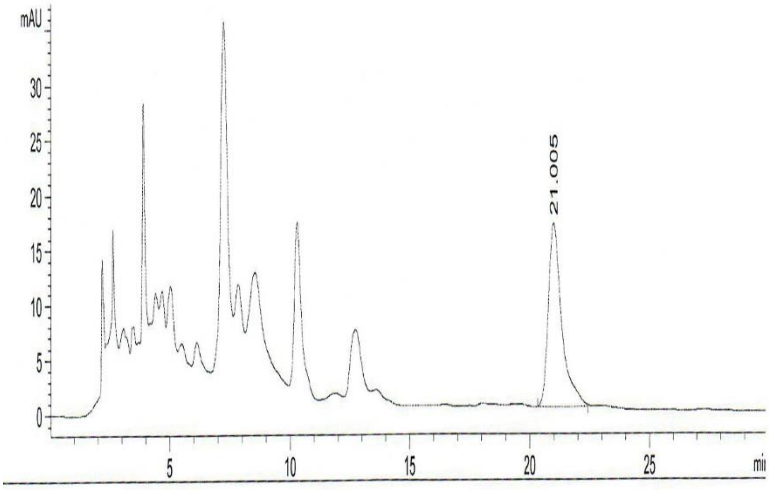

Figure. S5 The LC of *Ligusticum striatum DC.*

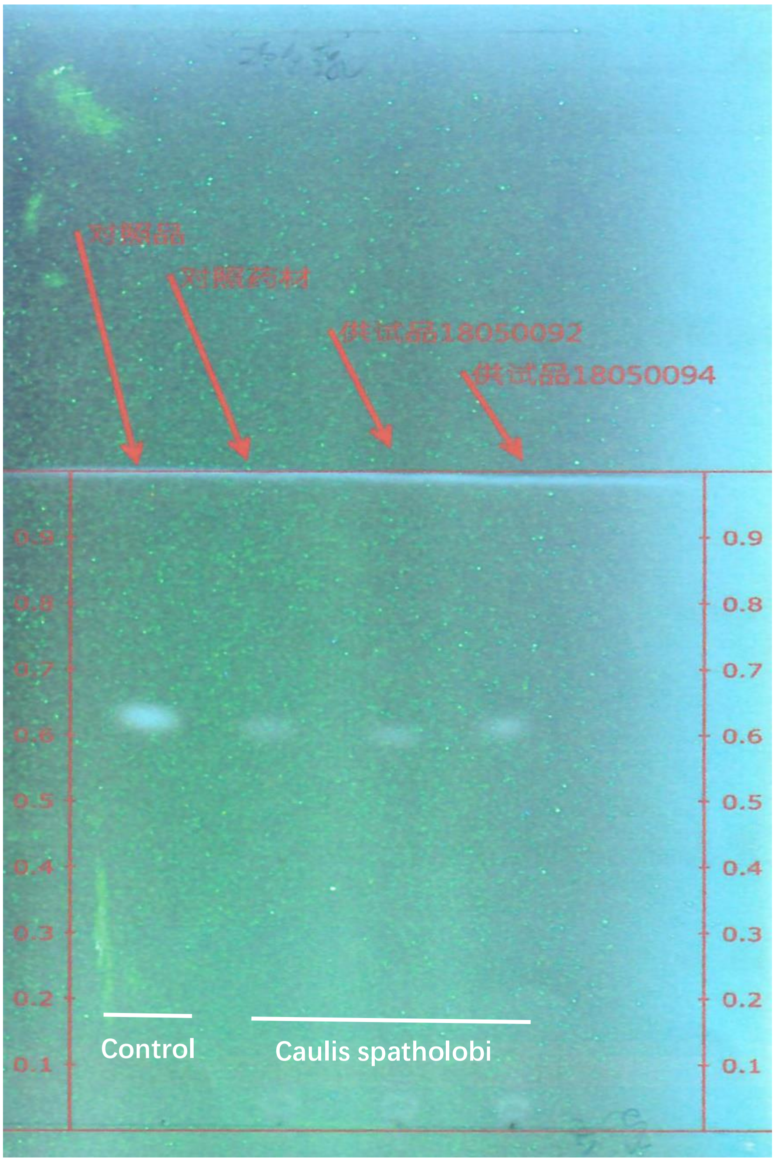

Figure. S6 The TLC of *Caulis spatholobi*.

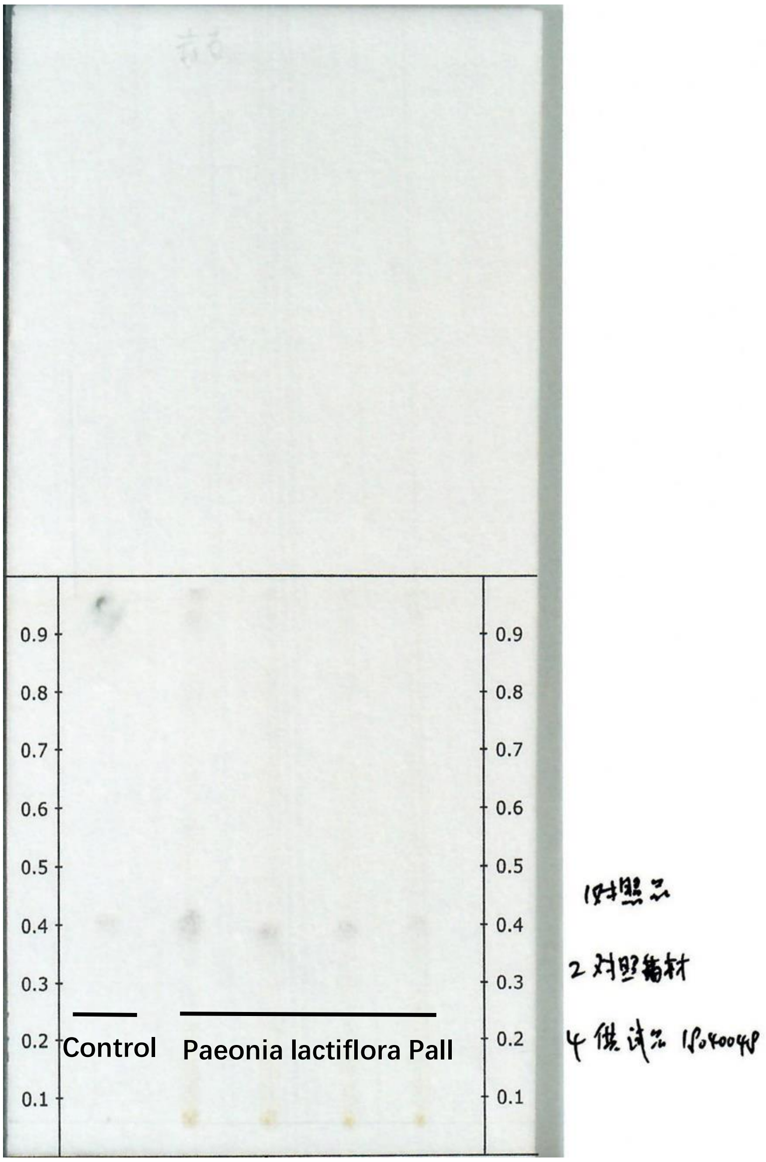

Figure. S7 The TLC of *Paeonia lactiflora Pall.*

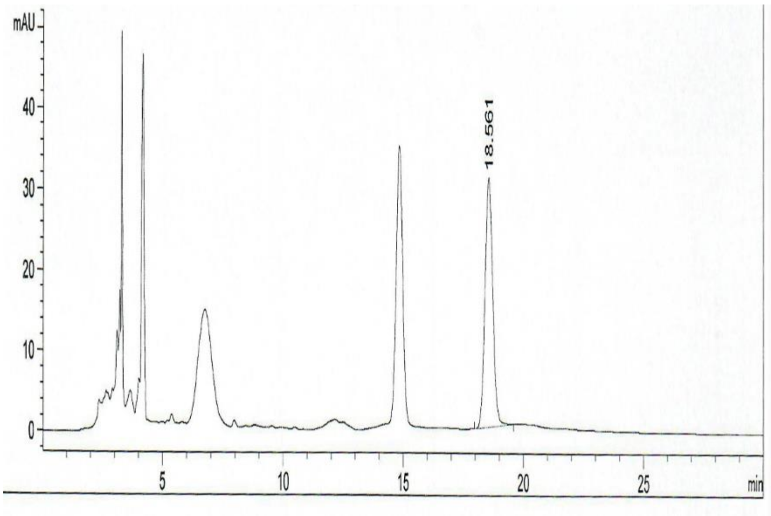

Figure. S8 The LC of *Paeonia lactiflora Pall.*

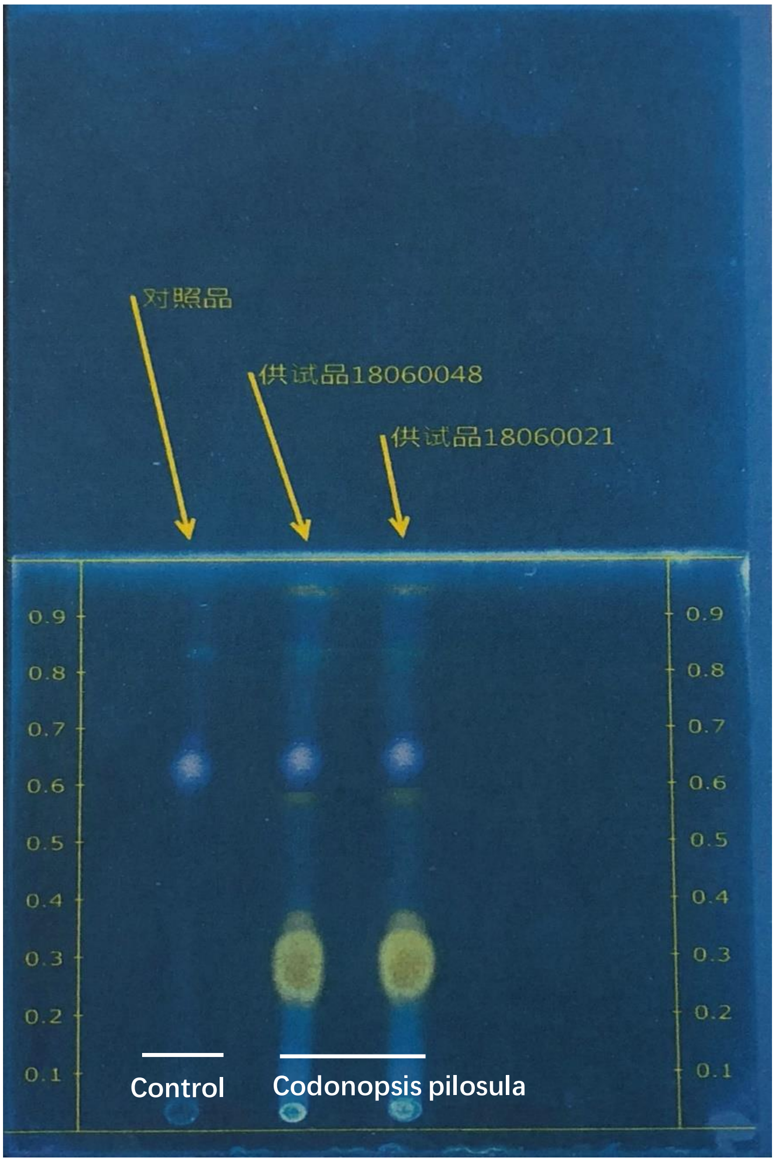

Figure. S9 The TLC of *Codonopsis pilosula (Franch.)*.
